# The Biological Role of Nestin^(+)^-Cells in Physiological and Pathological Cardiovascular Remodeling

**DOI:** 10.3389/fcell.2018.00015

**Published:** 2018-02-14

**Authors:** Angelino Calderone

**Affiliations:** ^1^Département de Pharmacologie et Physiologie, Université de Montréal, Montréal, QC, Canada; ^2^Montreal Heart Institute, Montréal, QC, Canada

**Keywords:** nestin, heart, embryogenesis, neural progenitor/stem cells, fibroblasts, cardiomyocytes, p38 MAPK, vasculature

## Abstract

The intermediate filament protein nestin was identified in diverse populations of cells implicated in cardiovascular remodeling. Cardiac resident neural progenitor/stem cells constitutively express nestin and following an ischemic insult migrate to the infarct region and participate in angiogenesis and neurogenesis. A modest number of normal adult ventricular fibroblasts express nestin and the intermediate filament protein is upregulated during the progression of reparative and reactive fibrosis. Nestin depletion attenuates cell cycle re-entry suggesting that increased expression of the intermediate filament protein in ventricular fibroblasts may represent an activated phenotype accelerating the biological impact during fibrosis. Nestin immunoreactivity is absent in normal adult rodent ventricular cardiomyocytes. Following ischemic damage, the intermediate filament protein is induced in a modest population of pre-existing adult ventricular cardiomyocytes bordering the peri-infarct/infarct region and nestin^(+)^-ventricular cardiomyocytes were identified in the infarcted human heart. The appearance of nestin^(+)^-ventricular cardiomyocytes post-myocardial infarction (MI) recapitulates an embryonic phenotype and depletion of the intermediate filament protein inhibits cell cycle re-entry. Recruitment of the serine/threonine kinase p38 MAPK secondary to an overt inflammatory response after an ischemic insult may represent a seminal event limiting the appearance of nestin^(+)^-ventricular cardiomyocytes and concomitantly suppressing cell cycle re-entry. Endothelial and vascular smooth muscle cells (VSMCs) express nestin and upregulation of the intermediate filament protein may directly contribute to vascular remodeling. This review will highlight the biological role of nestin^(+)^-cells during physiological and pathological remodeling of the heart and vasculature and discuss the phenotypic advantage attributed to the intermediate filament protein.

## Introduction

Cardiovascular remodeling secondary to an ischemic insult or a chronic hemodynamic overload involves the interplay of numerous biological events driven by diverse cell populations. A seminal phenotype of several cell populations is the increased expression or *de novo* synthesis of the intermediate filament protein nestin secondary to a pathological stress. The normal adult rodent heart contains a resident population of neural progenitor/stem cells that constitutively express nestin. A paucity of normal adult ventricular fibroblasts expresses nestin and the intermediate filament protein is upregulated during the progression of reactive and reparative fibrosis. Nestin is absent in normal adult rodent ventricular cardiomyocytes but following ischemic damage the intermediate filament protein is induced *de novo* in a modest population identified predominantly at the peri-infarct/infarct region. These findings are translatable to the clinical setting as interstitial and scar-residing nestin^(+)^-cells and a population of nestin^(+)^-cardiomyocytes were identified in the heart of post-myocardial infarcted patients. Nestin upregulation also represents an important feature of vascular remodeling and the intermediate filament protein was further identified in human endothelial and vascular smooth muscle cells (VSMCs). The present review will highlight the biological role of nestin^(+)^-cells during physiological and pathological cardiovascular remodeling and discuss the biological impact of the intermediate filament protein.

## Reparative fibrosis and angiogenesis; scar formation and healing of the ischemically damaged adult mammalian heart

**Figure 1:**
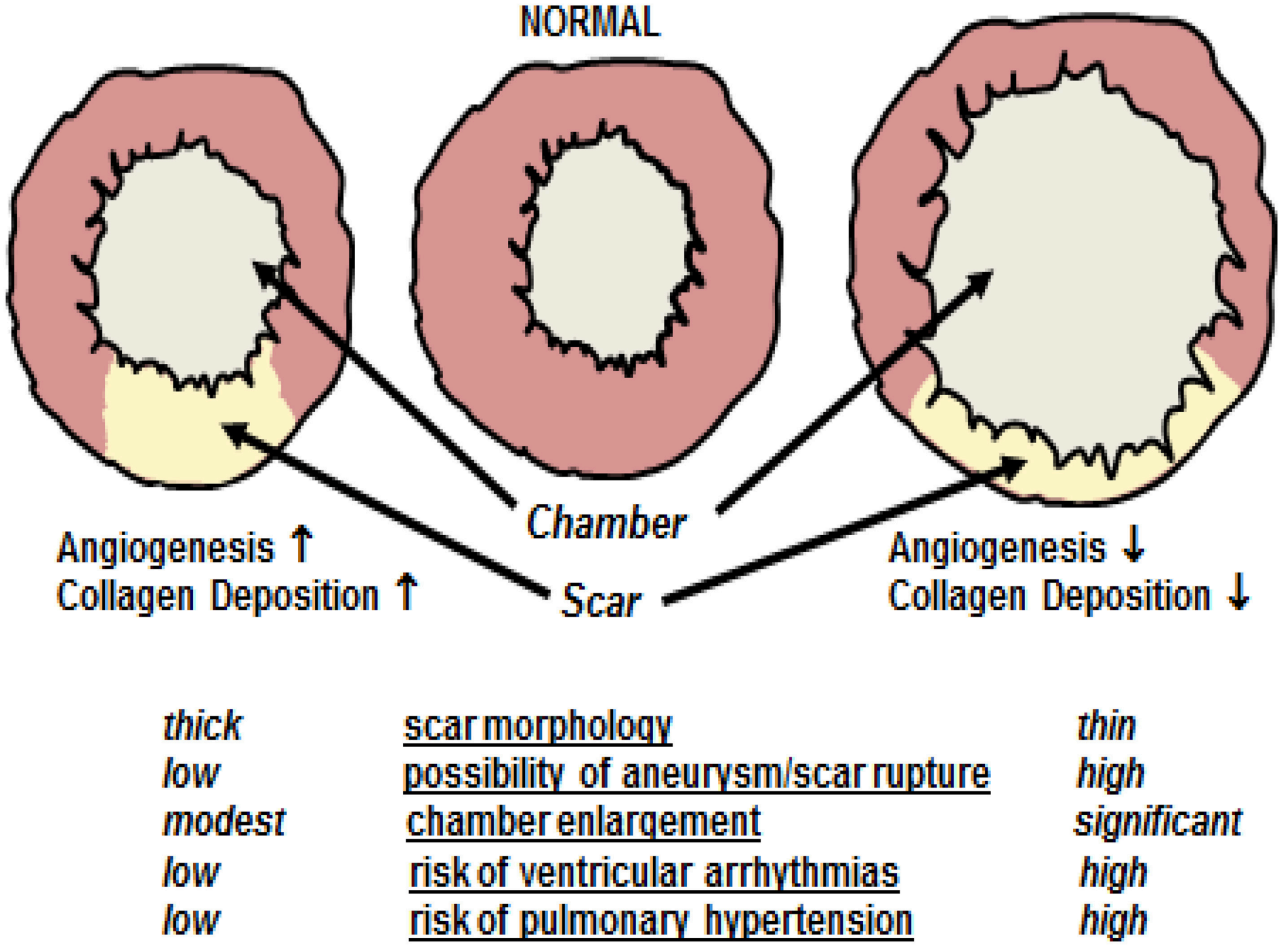
Cardiac remodeling following myocardial infarction. A compromised angiogenic response and/or reduced deposition of collagen type I secondary to a diminished recruitment and/or proliferation of myofibroblasts leads to inadequate scar formation characterized by infarct thinning. Inadequate scar formation exacerbates left ventricular dilatation characterized by chamber enlargement. In some rare cases, significant scar thinning could lead to cardiac rupture and death. Furthermore, left ventricular dilatation is as a negative prognostic factor in heart failure patients associated with an increased incidence of ventricular arrhythmias and development of pulmonary hypertension. By contrast, robust reparative fibrotic, and angiogenic responses leads to scar thickening thereby limiting chamber enlargement post-myocardial infarction and concomitantly reduces the risks associated with left ventricular dilatation.

Ischemic injury of the adult mammalian heart leads to an overt inflammatory response characterized by the recruitment of neutrophils and monocyte-derived macrophages to the damaged region leading to the phagocytosis of necrotic tissue (Chen and Frangogiannis, 2013; Prabhu and Frangogiannis, 2016). As repair proceeds, cytokines (e.g., tumor necrosis factor-α, interleukin-1β, and transforming growth factor-β) released by invading pro-inflammatory cells initiates the recruitment of ventricular fibroblasts from the non-infarcted left ventricle (NILV) to the ischemic area and concomitantly induces differentiation to a myofibroblast phenotype (Chen and Frangogiannis, 2013; Prabhu and Frangogiannis, 2016). In contrast to normal adult ventricular fibroblasts, myofibroblasts are characterized by smooth muscle α-actin expression and secrete greater amounts of the extracellular matrix protein collagen to rapidly heal the ischemically damaged heart (Chen and Frangogiannis, 2013; Prabhu and Frangogiannis, 2016). The process of scar formation/healing denoted as reparative fibrosis represents an essential physiological event repairing the ischemically damaged heart in the absence of ventricular regeneration. Physiologically, the scar provides needed structural support limiting left ventricular dilatation of the ischemically damaged heart (Figure 1; Ahmad et al., 2014; Richardson and Holmes, 2015; Iyer et al., 2016). A compromised proliferative response and/or diminished recruitment of myofibroblasts associated with a concomitant reduction of collagen deposition leads to infarct thinning exacerbating left ventricular dilation and in some rare cases could result in cardiac rupture and death (Figure 1; Trueblood et al., 2001; Dai et al., 2005; Shimazaki et al., 2008; Sun et al., 2011; Van Aelst et al., 2015). Clinically, left ventricular dilatation was identified as a negative prognostic factor in heart failure patients associated with an increased incidence of ventricular arrhythmias and development of pulmonary hypertension (Figure 1; Jasmin et al., 2003; Weintraub et al., 2017).

The *de novo* appearance of blood vessels in the peri-infarct/infarct region during scar formation/healing of the ischemically damaged adult mammalian heart is defined as reparative angiogenesis. A robust reparative angiogenic response is associated with a smaller scar region attributed in part to a reduction in the apoptotic loss of cardiomyocytes and a concomitant thicker infarct secondary to the increased deposition of collagen by resident myofibroblasts (Figure 1) (Barandon et al., 2003; Fazel et al., 2006; Cochain et al., 2013). Moreover, myofibroblasts synthesize vascular endothelial growth factor-A (VEGF-A) and acting via a paracrine fashion may further potentiate the *de novo* appearance of blood vessels during reparative fibrosis (Chintalgattu et al., 2007; El-Helou et al., 2012; Lin et al., 2012). Thus, in the absence of ventricular regeneration, reparative angiogenesis restricts infarct expansion, and promotes scar thickening thereby limiting left ventricular dilatation (Figure 1). The apparent interaction between angiogenesis and fibrosis is not exclusive to the heart as the appearance of *de novo* blood vessels in damaged skin is essential for the ensuing fibrotic response initiated by invading myofibroblasts leading to hypertrophic scarring (Eming et al., 2014).

## Distinct introns of the nestin gene drive expression in cell specific manner

The 240-kDa protein nestin is a member of the class VI family of intermediate filament proteins and in contrast to other classes, is unable to self-assemble and form homodimers because of a short N-terminus (Frederiksen et al., 1988; Lendahl et al., 1990; Wiese et al., 2004; Neradil and Veselska, 2015). Thus, depending on the cell type, nestin will form heterodimers with other intermediate filament proteins including vimentin, α-internexin, and desmin (Wiese et al., 2004; Neradil and Veselska, 2015). The human and rat nestin gene possess three conserved intron regions that drive expression of the intermediate filament protein in a cell specific manner (Lothian et al., 1999; Wiese et al., 2004; Neradil and Veselska, 2015). Nestin expression in neural stem cells of the central nervous system (CNS) is independently regulated by two distinct enhancer elements identified in the second intron of the gene (Lendahl et al., 1990; Zimmerman et al., 1994; Lothian et al., 1999; Yaworsky and Kappen, 1999; Mignone et al., 2004). By contrast, intron 1 of the nestin gene selectively drives expression of the intermediate filament protein in endothelial cells and skeletal muscle (Zimmerman et al., 1994; Aihara et al., 2004; Zhong et al., 2008).

## Neural crest-derived neural progenitor/stem cells that express nestin identified in the adult rodent heart; biological role during reparative angiogenesis

**Figure 2:**
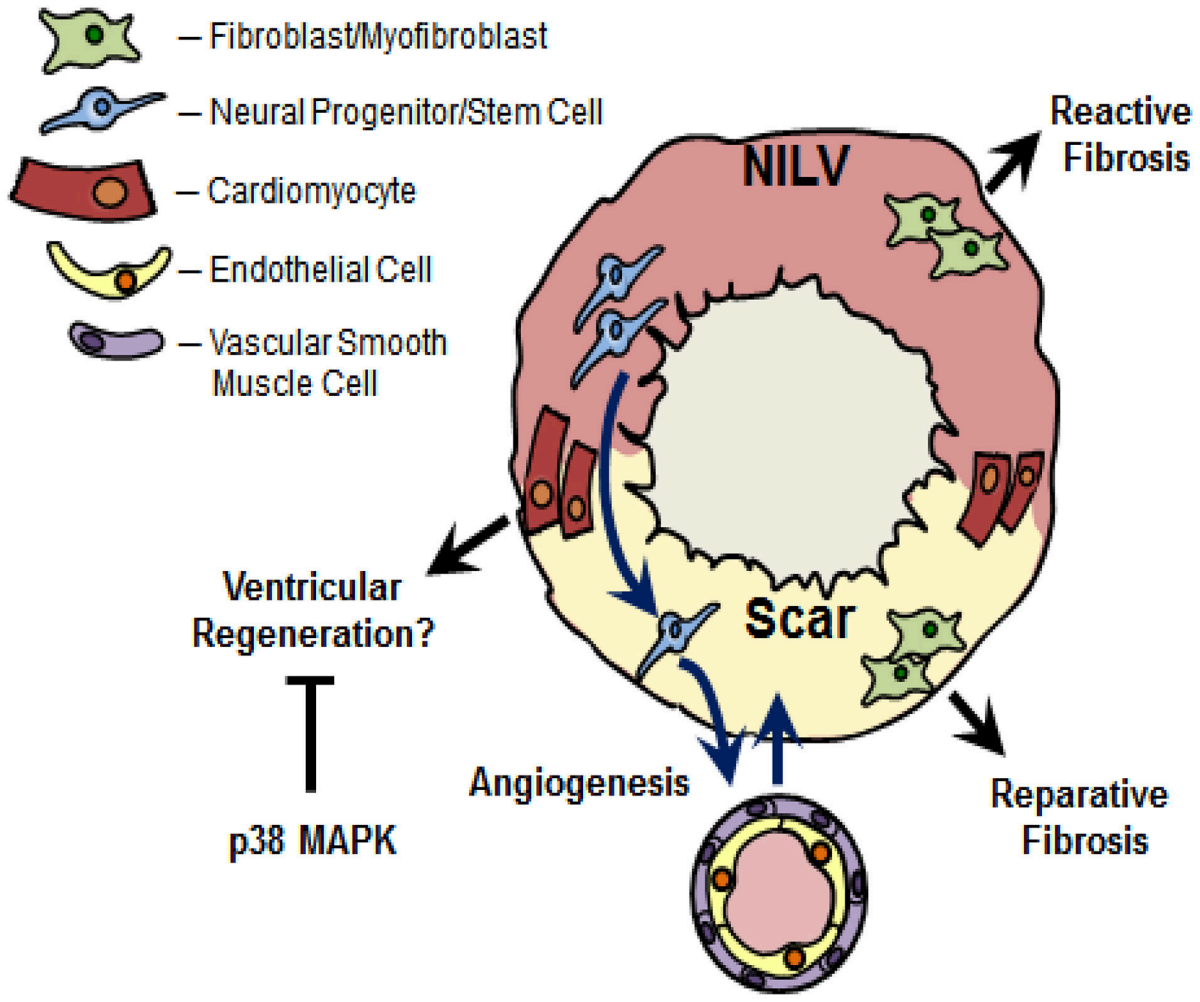
Nestin^(+)^-cells and cardiac remodeling following myocardial infarction. Ischemic injury to the adult rodent heart leads to the migration of cardiac resident nestin^(+)^-neural progenitor/stem cells from the non-infarcted left ventricle (NILV) to the scar region and subsequent differentiation to a vascular cell leading to *de novo* blood vessel formation. A subpopulation of cardiac resident nestin^(+)^-neural progenitor/stem cells also participate in the neurogenic response during scar formation (not depicted in figure). Recapitulation of the intermediate filament protein in scar-residing myofibroblasts may represent an activated phenotype to rapidly heal the infarct region during reparative fibrosis. The increased appearance of nestin^(+)^-fibroblasts was also reported in the fibrotic heart secondary to pressure-overload, in fibrotic lungs secondary to hypobaric hypoxia and the fibrotic kidney following unilateral ureteral obstruction. In this regard, the increased denisty of nestin^(+)^-fibroblasts may play a seminal role driving the reactive fibrotic response, regardless the tissue. Lastly, nestin was re-expressed in pre-existing adult cardiomyocytes detected predominantly at the peri-infarct/infarct region of the ischemically damaged heart. *In vitro* data revealed that nestin expression drives the cell cycle re-entry of neonatal rat ventricular cardiomyocytes. Collectively these data suggest that the appearance of nestin^(+)^-cardiomyocytes in the adult mammalian infarcted heart may represent an inherent paradigm of ventricular regeneration. Previous studies have reported that p38 MAPK inhibits the cell cycle re-entry and subsequent cytokinesis of ventricular cardiomyocytes. Based on the latter data, the modest appearance of nestin^(+)^-cardiomyocytes and concomitant inhibition of cell cycle re-entry may be likewise attributed in part to a suppressive action of p38 MAPK recruited by the overt inflammatory response post-myocardial infarction.

Stem cells derived from the neural crest migrate throughout the developing embryo and differentiate to diverse cell types including sympathetic neurons, glial cells, chondrocytes, epithelium, and VSMCs (Sieber-Blum and Grim, 2004; Dupin and Le Douarin, 2014; Plein et al., 2015). During cardiogenesis, neural crest stem cells participate in the septation of the cardiac outflow tract into the pulmonary artery and aorta (Dupin and Le Douarin, 2014; Plein et al., 2015). Following postnatal development, several studies have reported that a residual population of neural crest-derived stem cells persist in the heart and skin (Fernandes et al., 2004, 2008; Drapeau et al., 2005; Tomita et al., 2005; El-Helou et al., 2008). Work from our lab and Fukuda's group independently identified a resident neural crest-derived population of cells in the adult rodent heart that express the intermediate filament protein nestin and exhibit a neural progenitor/stem cell phenotype (Table 1; Figure 2) (Drapeau et al., 2005; El-Helou et al., 2005, 2008; Tomita et al., 2005). The labs of Freda Miller and Robert Hoffman likewise reported that hair follicles of the adult rodent represent a niche of neural crest-derived cells that express nestin and display a neural progenitor/stem cell phenotype (Toma et al., 2001; Amoh et al., 2004; Fernandes et al., 2004, 2008). Moreover, hair follicle- and cardiac-resident neural progenitor/stem cells retain expression of several neural crest related transcriptional factors including sox9 and nestin expression is driven by intron 2 of the gene (Li et al., 2003; Tomita et al., 2005; El-Helou et al., 2008; Fernandes et al., 2008; Table 1). Following isolation, neural progenitor/stem cells (regardless the tissue) grow as floating spheres *in vitro* in the presence of epidermal growth factor/basic fibroblast growth factor (EGF/bFGF) and differentiate to a neuronal/glial cell in the presence of defined stimuli (Tomita et al., 2005; Fernandes et al., 2008; El-Helou et al., 2009). In the normal adult rodent heart, neural crest-derived nestin^(+)^-neural progenitor/stem cells are intercalated between cardiomyocytes and following myocardial infarction (MI) migrate to the scar region (El-Helou et al., 2005, 2008; Tamura et al., 2011). Nestin^(+)^-cells were also identified in the non-infarcted myocardium and scar region of the ischemically damaged adult human heart, albeit it remains presently unknown if a subpopulation represent neural progenitor/stem cells (El-Helou et al., 2008).

**Table 1:** Nestin^(+)^-cells implicated in cardiovascular remodeling.

**Cell type**	**Nestin**	**Intron**	**Stimulus**	**Biological role**
Cardiac resident neural progenitor/Stem cells	Constitutive	Intron 2	Myocardial infarction	Reparative fibrosis (angiogenesis and neurogenesis) (Tomita et al., 2005; El-Helou et al., 2008, 2013; Béguin et al., 2011; Chabot et al., 2013)
Pre-existing adult cardiomyocytes	Induced *de novo*	Intron 1 or 3	Myocardial infarction	Ventricular regeneration? (cell cycle re-entry) (Meus et al., 2017; Hertig et al., 2018)
Adult ventricular fibroblasts	Constitutive and upregulated	Intron 1 or 3	Myocardial infarction and hypertension	Reparative and reactive fibrosis (proliferation) (Béguin et al., 2012; Hertig et al., 2017)
Endothelial cells	Constitutive and upregulated	Intron 1	Myocardial infarction and hypertension	Angiogenesis (proliferation and migration) (Mokrý et al., 2004, 2008; Liang et al., 2015)
Displaced endothelial cells (interstitial)	Unknown	Intron 1?	Hypertension	Reactive fibrosis? (Hertig et al., 2017)
Perivascular Nestin^(+)^-cells (lack SMA and collagen)	Unknown	Unknown	Hypertension	Perivascular fibrosis? (Hertig et al., 2017)
Vascular smooth muscle cells	Constitutive and upregulated	Unknown	Hypertension	Vessel remodeling (proliferation) (Oikawa et al., 2010; Tardif et al., 2014, 2015)

*Table summarizes the diverse populations of nestin^(+)^-cells identified in the adult mammalian heart and vasculature, highlights whether nestin is constitutively expressed and subsequently upregulated or induced de novo, identifies the intron region and the stimuli influencing expression of the intermediate filament protein in the heart or vasculature and biological role during tissue remodeling. In displaced endothelial cells, intron 1 may regulate nestin expression but definitive data is lacking. Furthermore, the biological role of displaced nestin/collagen type I^(+)^-endothelial cells during reactive fibrosis remains to be elucidated. The cellular source and functional role of nestin^(+)^-cells (lack smooth muscle α-actin and collagen expression) bordering the perivascular fibrotic region of predominantly large caliber coronary blood vessels of the pressure-overloaded rat heart remain undefined. The references cited represent the seminal papers identifying the presence and/or biological role of the various populations of nestin^(+)^-cells implicated in cardiovascular remodeling*.

In 2004, Wurmser et al. revealed that a subpopulation of CNS-resident neural stem cells co-cultured with endothelial cells in matrigel acquire an endothelial cell phenotype and reorganize into vascular structures (Wurmser et al., 2004). An analogous response was observed after the transplantation of neural stem cells in the telencephalon of embryonic mice (Wurmser et al., 2004). However, the percentage of transplanted neural progenitor/stem cells that differentiated to an endothelial cell leading to a vascular structure was significantly lower than that observed *in vitro* (Wurmser et al., 2004). An important angiogenic response may be prevalent during pathological remodeling as vascularisation of glioblastomas was reported to occur in part via the differentiation of cancer-like neural stem cells to an endothelial cell phenotype (Ricci-Vitiani et al., 2010). Consistent with the vascular plasticity of CNS-resident neural stem cells, skin-derived neural progenitor/stem cells located within the bulge region of the hair follicle form capillary networks after wound healing (Aki et al., 2010). These observations provided the impetus to examine whether cardiac resident neural progenitor/stem cells represent a novel cellular substrate of reparative angiogenesis during scar formation/healing of the infarcted adult rodent heart. Cardiac resident neural progenitor/stem cells were isolated from the scar of 1 week post-MI rats, grown as spheres in the presence of EGF/bFGF and subsequently labeled with a chromophore to track migration and differentiation after transplantation in the NILV of the infarcted rat heart (El-Helou et al., 2008). Transplanted cardiac neural progenitor/stem cells migrated to the scar and a subpopulation preferentially differentiated to an endothelial cell co-expressing endothelial nitric oxide synthase (eNOS) and nestin leading to the *de novo* appearance of predominantly small caliber blood vessels (Table 1; Figure 2) (El-Helou et al., 2008). A modest number of transplanted neural progenitor/stem cells identified in newly formed vascular structures differentiated a VSMC phenotype (Table 1; Figure 2) (El-Helou et al., 2008). In support of the latter finding, CNS-derived embryonic neural stem cells cultured in the presence of the appropriate stimulus likewise acquired a VSMC phenotype *in vitro* (Oishi et al., 2002). To reaffirm the angiogenic potential of cardiac resident neural progenitor/stem cells, the heart of adult transgenic mice expressing the reporter enhanced green fluorescent protein (EGFP) driven by intron 2 of the nestin gene was subjected to complete occlusion of the left anterior descending coronary artery. In the heart of normal adult transgenic mice not subjected coronary artery occlusion, EGFP was co-expressed in nestin^(+)^-neural progenitor/stem cells detected intercalated between cardiomyocytes (El-Helou et al., 2013). Native blood vessels identified in the normal left ventricle lacked EGFP immunoreactivity albeit nestin staining was detected in endothelial cells (El-Helou et al., 2013). The latter data is consistent with the observation that intron 1 rather than intron 2 of the nestin gene drives expression of the intermediate filament protein in endothelial cells (Aihara et al., 2004). Consistent with the findings of our previous study (El-Helou et al., 2008), endothelial cells identified predominantly in small caliber blood vessels of the scar region were derived in part from differentiated resident neural progenitor/stem cells characterized by the co-expression of nestin, CD31, and the reporter EGFP (Table 1; Figure 2) (El-Helou et al., 2013; Meus et al., 2017). In addition, a modest number of scar-residing blood vessels were delineated by EGFP staining of smooth muscle α-actin^(+)^-VSMCs (Table 1; Figure 2) (Meus et al., 2017). Nestin-immunoreactive endothelial cells lacking EGFP staining were also identified in the infarct region suggesting that expression of the intermediate filament protein apparently represents a conserved phenotype of *de novo* blood vessel formation, regardless the cellular source (Mokrý et al., 2004; El-Helou et al., 2013; Meus et al., 2017). Collectively, these data reveal that cardiac resident neural progenitor/stem cells represent a novel cellular substrate of reparative angiogenesis during the progression of scar formation/healing following MI. Moreover, based on the importance of the reparative angiogenic response, a compromised neural progenitor/stem cell population could lead to maladaptive infarct remodeling exacerbating left ventricular dilatation. In type I and II diabetes, nestin protein levels were significantly diminished in cardiac resident neural progenitor/stem cells (El-Helou et al., 2009). The loss of nestin would have a profound biological impact as the intermediate filament protein directly facilitates the proliferation and migration of neural progenitor/stem cells (Xue and Yuan, 2010; Yan et al., 2016). A compromised proliferative and migratory phenotype of neural progenitor/stem cells secondary to loss of nestin may have contributed in part to the impaired angiogenic response documented after ischemic injury to the heart and damage to the skin predisposed to a diabetic environment (Ikeda et al., 2012; Wang et al., 2015; Okonkwo and DiPietro, 2017).

The conserved angiogenic plasticity of neural progenitor/stem cells provided the impetus to test the hypothesis that transplantation of isogenic skin-derived neural progenitor/stem cells into the infarcted adult heart would lead to a smaller scar secondary to the increased availability of cellular substrate for *de novo* blood vessel formation (El-Helou et al., 2013). Skin-derived neural progenitor/stem cells isolated from embryonic Sprague-Dawley rats expressed the intermediate filament protein nestin, the neural crest-related transcriptional factors sox2 and sox9, and grew as spheres in the presence of EGF/bFGF (El-Helou et al., 2013). Following transplantation of the NILV of 3-day post-MI Sprague-Dawley rats, chromophore labeled skin-derived neural progenitor/stem cells were detected engrafted in the peri-infarct/infarct region. Despite migration and engraftment, skin-derived neural progenitor/stem cells failed to differentiate to a vascular phenotype (El-Helou et al., 2013). Nonetheless, a significant reduction of scar size and concomitant improvement of left ventricular function were observed in the infarcted heart transplanted with skin-derived neural progenitor/stem cells (El-Helou et al., 2013). The smaller infarct region was attributed to the increased density of predominantly small caliber blood vessels in the peri-infarct/infarct region facilitated in part by the paracrine action of a panel of pro-angiogenic peptide growth factors (e.g., VEGF-A, nerve growth factor, stromal cell-derived factor-1α) released by engrafted skin-derived neural progenitor/stem cells (El-Helou et al., 2013). Thus, despite important phenotypic similarities (e.g., nestin, sox9, growth as spheres) and inherent angiogenic plasticity of skin- and cardiac-derived neural progenitor/stem cells, the scar region of the infarcted adult heart did not represent a favorable environment of engrafted skin-derived neural progenitor/stem cells to differentiate to a vascular cell. A similar paradigm was observed following the engraftment of brain-derived neural progenitor/stem cells in the scar of a myocardial infarcted adult mouse, as differentiation to a vascular cell and subsequent *de novo* blood vessel formation was modest (Ii et al., 2009). These observations suggest that the environment of the damaged tissue rather than the inherent plasticity may represent the primary variable limiting and/or suppressing the differentiation of neural progenitor/stem cells to a vascular phenotype following transplantation.

## Neural progenitor/stem cells and the neurogenic response during reparative fibrosis

Sympathetic hyperinnervation characterized by an increased density of growth associated protein 43 (GAP43)-, neurofilament-M, and tyrosine hydroxylase^(+)^-fibers represents an established phenotypic event of the ischemically damaged adult mammalian heart providing inotropic support and initiating cardiomyocyte hypertrophy (Zhou et al., 2004; Hasan et al., 2006; Woodcock et al., 2008; Triposkiadis et al., 2009; Kreipke and Birren, 2015). However, sympathetic hyperinnervation post-MI also leads to maladaptive remodeling including the genesis of arrhythmias, infarct expansion, and reactive fibrosis (Cao et al., 2000; Colucci et al., 2000; Colombo et al., 2003; Li et al., 2015). Sympathetic remodeling of the ischemically damaged heart provided the impetus to examine whether a subpopulation of cardiac resident neural progenitor/stem cells contributes to the neurogenic response. Indeed, a subpopulation of neurofilament-M^(+)^-fibers innervating the peri-infarct/infarct region of the ischemically damaged adult rat heart were physically associated with nestin^(+)^-processes derived from cardiac resident neural progenitor/stem cells (Table 1; El-Helou et al., 2008; Béguin et al., 2011). By contrast, a physical association of nestin^(+)^-processes and neurofilament-M^(+)^-fibers was not observed in the normal adult rat heart or NILV of the infarcted rat heart (El-Helou et al., 2005, 2008). Unequivocal evidence of a neurogenic role of neural progenitor/stem cells was delineated in a rat model of cardiac transplantation (Béguin et al., 2011). Complete denervation characterized by the absence of innervating neurofilament-M^(+)^-fibers was achieved following isogenic heterotopic transplantation of an adult rat heart into the abdomen of a recipient rat (Béguin et al., 2011). Ischemic injury to the beating transplanted rat heart led to the *de novo* synthesis of neurofilament-M^(+)^-fibers physically associated with nestin^(+)^-processes emanating from neural progenitor/stem cells (Béguin et al., 2011). The neurogenic response of cardiac resident neural progenitor/stem cells post-MI is characterized by the initial expression of GAP43^(+)^-fibers physically associated with nestin^(+)^-processes and subsequently replaced by neurofilament-M^(+)^-fibers (Chabot et al., 2013). The temporal neurogenic response is consistent with previous studies revealing that GAP43 expression represents an early event of neurogenesis during learning and following regeneration of injured peripheral nerves (Leu et al., 2010; Grasselli et al., 2011). To delineate the stimuli implicated, normal adult rats were implanted with an osmotic pump containing nerve growth factor as the local production of the neurotrophin by cardiomyocytes, myofibroblasts, and invading macrophages was reported to play a seminal role driving sympathetic hyperinnervation post-MI (Zhou et al., 2004; Hasan et al., 2006). Osmotic pump delivery of nerve growth factor increased the density of neurofilament-M fibers innervating the heart, albeit a physical association with nestin^(+)^ processes was not observed (Béguin et al., 2011). Thus, the appearance and physical association of GAP43^(+)^- and neurofilament^(+)^-fibers with nestin^(+)^-processes emanating from a subpopulation of neural progenitor/stem cells was prevalent in the adult heart only after an ischemic insult. The identity of the stimulus locally synthesized/released in the scar mediating the neurogenic response of cardiac resident neural progenitor/stem cells is presently unknown and the biological impact on infarct remodeling remains to be elucidated.

## Reparative/reactive fibrosis and nestin expression in ventricular fibroblasts

It was originally assumed that nestin represents a selective marker of neural crest-derived cells that exhibit a neural progenitor/stem cell phenotype. However, numerous studies have reported that the intermediate filament protein is expressed in a wide variety of normal and tumorigenic cells (Oikawa et al., 2010; Ishiwata et al., 2011; Béguin et al., 2012; Liang et al., 2015; Neradil and Veselska, 2015). Among the various populations of cells expressing nestin, the intermediate filament protein was detected in ventricular fibroblasts and dynamically regulated during postnatal development and in the fibrotic heart secondary to an ischemic insult or pressure-overload (Béguin et al., 2012; Hertig et al., 2017). Nestin is ubiquitously expressed in neonatal rat ventricular fibroblasts and postnatal development led to a significant downregulation of the intermediate filament protein in adult rat ventricular fibroblasts (Béguin et al., 2012). Moreover, neonatal rat ventricular fibroblasts were associated with a higher basal rate of DNA synthesis and greater expression of the extracellular matrix protein collagen type I and the pro-fibrotic peptide growth factor transforming growth factor-β_3_ as compared to normal adult ventricular fibroblasts (Béguin et al., 2012; El-Helou et al., 2012). Following MI of the adult rat heart, nestin was upregulated in scar-residing myofibroblasts as a greater percentage expressed the intermediate filament protein as compared to normal adult ventricular fibroblasts (Figure 2) (Béguin et al., 2012; El-Helou et al., 2012). Consistent with these data, Scobioala et al. (2008) reported that nearly 50% of discoidin domain receptor 2-^(+)^-ventricular fibroblasts identified in the peri-infarct/infarct region were nestin immunoreactive and the percentage was significantly greater than that observed in the non-infarcted myocardium. Employing adult transgenic mice in which intron 2 of the nestin gene drives EGFP expression, the reporter was not detected in nestin/smooth muscle α-actin^(+)^-myofibroblasts identified in the peri-infarct/infarct region (Meus et al., 2017). Thus, in contrast to neural progenitor/stem cells, intron 1 and/or intron 3 of the nestin gene drives expression of the intermediate filament protein in scar-residing myofibroblasts (Table 1). Furthermore, the higher percentage of nestin^(+)^-myofibroblasts reported in the peri-infarct/infarct region was associated with a greater basal rate of DNA synthesis and expression of collagen type I and transforming growth factor-β_3_ as compared to normal adult ventricular fibroblasts (Béguin et al., 2012; El-Helou et al., 2012). Collectively, these data provided the impetus to examine whether the apparent dissimilar basal rate of DNA synthesis between neonatal fibroblasts, adult fibroblasts, and myofibroblasts was attributed in part to the disparate level of nestin expression. Indeed, the selective depletion of the intermediate filament protein following the infection of neonatal rat ventricular fibroblasts with a lentivirus containing a shRNA directed against nestin significantly reduced basal DNA synthesis (Béguin et al., 2012). Thus, postnatal development of rat ventricular fibroblasts is characterized by a significant downregulation of nestin protein levels and re-expression of the intermediate filament protein in myofibroblasts recapitulates an embryonic/neonatal phenotype. The enhanced proliferative response of nestin^(+)^-myofibroblasts may represent an activated pro-fibrotic phenotype to accelerate scar formation and healing (Scobioala et al., 2008; Béguin et al., 2012).

In contrast to the established physiological role of reparative fibrosis post-MI, reactive or interstitial fibrosis represents a pathophysiological response characterized by the uncontrolled synthesis/deposition of collagen type I by resident ventricular fibroblasts in the hypertrophied heart secondary to a chronic pressure-overload (e.g., hypertension) or in the hypertrophied NILV following ischemic damage (Figure 2) (Chen and Frangogiannis, 2013; Prabhu and Frangogiannis, 2016). In contrast to reparative fibrosis, reactive fibrosis reduces ventricular compliance thereby exacerbating systolic/diastolic dysfunction (Creemers and Pinto, 2011; Chen and Frangogiannis, 2013; Prabhu and Frangogiannis, 2016). An additional pathological feature of the pressure-overloaded heart is perivascular fibrosis characterized by the deposition of collagen type I within the adventitial region of the coronary vasculature leading to vascular stiffness (Dong et al., 2017). In the hypertrophied/fibrotic left ventricle of the rat heart secondary to pressure-overload, nestin protein levels were upregulated and attributed in part to the increased density of collagen type I-immunoreactive mesenchymal cells co-expressing the intermediate filament protein (Hertig et al., 2017). The increased density of nestin/collagen^(+)^-mesenchymal cells in the pressure-overloaded heart may be secondary to the upregulation of the intermediate filament protein in normal adult ventricular fibroblasts following exposure to the pro-fibrotic peptide growth factors angiotensin II, transforming growth factor-β_1_, and EGF (Hertig et al., 2017). An analogous paradigm was identified in the lungs secondary to hypobaric hypoxia as the reactive fibrotic response was associated with an increased density of collagen type 1-immunoreactive mesenchymal cells co-expressing nestin (Chabot et al., 2016). Consistent with the aforementioned studies, the overt accumulation of collagen in the fibrotic kidney following unilateral ureteral obstruction positively correlated with the density of nestin^(+)^-mesenchymal cells (Sakairi et al., 2007). Lastly, epithelial-to-mesenchymal transition was identified as a seminal event of reactive fibrosis in the lungs and a recent study revealed that lung epithelial cell transition to a mesenchymal phenotype was characterized by the loss of E-cadherin and concomitant upregulation of collagen type I and nestin (Kage and Borok, 2012; Chabot et al., 2016). Thus, akin to the paradigm identified during reparative fibrosis, the increased density of nestin/collagen type 1^(+)^-ventricular fibroblasts may represent a seminal event driving the reactive fibrotic response of the pressure-overloaded rat heart (Figure 2) (Table 1). These findings further reveal that the underlying nature of the insult to the adult mammalian heart rather than the conserved upregulation of nestin ultimately determines whether activated ventricular fibroblasts contribute to pathological (e.g., reactive fibrosis secondary to hypertension or in the NILV) or physiological (e.g., reparative fibrosis; scar formation) remodeling (Table 1). Lastly, a population of nestin^(+)^-cells lacking collagen type I expression was preferentially detected bordering the perivascular fibrotic region of predominantly large caliber coronary blood vessels of the pressure-overloaded rat heart (Hertig et al., 2017). The cellular source and potential contribution of this specific population of perivascular nestin^(+)^-cells in the progression of vascular fibrosis remains presently unresolved (Table 1).

## Nestin and vascular remodeling

In contrast to neural progenitor/stem cells, intron 1 of the nestin gene drives expression of the intermediate filament protein in endothelial cells and upregulation and/or induction of the intermediate filament protein identifies an angiogenic response during physiological and pathological remodeling (Table 1) (Aihara et al., 2004). Nestin was detected in endothelial cells lining ventricular trabeculae of the embryonic mouse heart, in newly formed blood vessels in the scar of the infarcted rodent and human heart and in the vasculature of fibrotic lungs secondary to hypobaric hypoxia (Mokrý et al., 2004, 2008; Wagner et al., 2006; El-Helou et al., 2009; Chabot et al., 2016; Hertig et al., 2018). Akin to that observed in ventricular and lung fibroblasts, transforming growth factor-β_1_ and EGF stimulation of rat microvascular endothelial cells induced nestin expression (Hertig et al., 2017). The exposure of human umbilical vein endothelial cells to a hypoxic environment promoted nestin expression and the intermediate filament protein directly facilitated VEGF-A mediated proliferation and migration during the formation of capillary-like tubules when plated in matrigel (Table 1; Liang et al., 2015). In the hypertrophied/fibrotic rat heart secondary to pressure-overload, displaced endothelial cells characterized by CD31 staining were unexpectedly detected in the interstitial milieu and co-expressed nestin and collagen type I (Table 1) (Hertig et al., 2017). By contrast, collagen type I staining was undetected in CD31/nestin^(+)^-endothelial cells identified in the coronary vasculature of the pressure-overloaded heart (Hertig et al., 2017). *In vitro* experiments reaffirmed the mesenchymal phenotype of displaced endothelial cells as CD31/eNOS^(+)^-rat microvascular endothelial cells expressed prolyl 4-hydroxylase A3 and collagen type I and secretion of the extracellular matrix protein led to the formation of a fibrillary network (Hertig et al., 2017). A mesenchymal phenotype was also identified in endothelial cells isolated from the cornea and fibrotic liver and work from our lab reported that CD31/eNOS^(+)^-human coronary artery endothelial cells express collagen type I and filamentous nestin (Howard et al., 1976; Maher and McGuire, 1990; Hertig et al., 2017). These data collectively suggest that interstitial nestin/CD31^(+)^-endothelial cells identified in the pressure-overloaded rat heart do not undergo endothelial-mesenchymal transformation but rather displacement from the vasculature unmasks a mesenchymal phenotype. Despite these findings, the contribution of displaced interstitial nestin/CD31/collagen type 1^(+)^-endothelial cells to the reactive fibrotic response of the pressure-overloaded adult rat heart remains presently unresolved (Table 1).

Oikawa et al. (2010) reported that nestin was expressed in VSMCs of the normal adult rat aorta and the density of nestin^(+)^-VSMCs in the aortic arch was significantly greater as compared to the thoracic and abdominal regions (Table 1). Data from our lab reaffirmed the latter paradigm and further demonstrated that a subpopulation of nestin^(+)^-VSMCs of the adult rat aorta were in G2-M phase as depicted by phosphohistone-3 staining (Tardif et al., 2014, 2015). Nestin^(+)^-VSMCs co-expressing phosphohistone-3 were also detected in the adult rat carotid artery and the intermediate filament protein was further identified in endothelial cells lining the aorta and carotid artery (Tardif et al., 2014, 2015). Consistent with the *in vivo* data, VSMCs isolated from the rat carotid artery and aorta characterized by the smooth muscle α-actin, smooth muscle-22α, and caldesmon immunoreactivity co-expressed filamentous nestin (Tardif et al., 2015). Likewise, filamentous nestin was detected in human aortic-derived caldesmon/smooth muscle-22α^(+)^-vascular smooth muscle cells and VSMCs of the pulmonary vasculature of heart failure patients (Chabot et al., 2015; Tardif et al., 2015). Exposure of adult rat carotid artery- and aortic-vascular smooth muscle cells to a panel of peptide growth factors upregulated nestin protein levels (Tardif et al., 2015). Nestin protein depletion of rat carotid artery VSMCs via a lentiviral-shRNA approach significantly attenuated angiotensin II and EGF mediated DNA synthesis, whereas no change in protein synthesis was observed (Table 1) (Tardif et al., 2015). These findings provided the impetus to determine whether vessel remodeling secondary to a hypertensive state would lead to upregulation of nestin in VSMCs. Indeed, secondary to pressure-overload (e.g., suprarenal abdominal aorta constriction), adult rat carotid artery and aorta vessel remodeling (↑ wall thickness and media area) was associated with a significant increase in nestin protein levels (Tardif et al., 2015). Moreover, nestin expression in the carotid artery of hypertensive rats positively correlated with the rise of mean arterial pressure and left ventricular systolic pressure and attributed in part to a greater density of nestin^(+)^-vascular smooth muscle cells as compared to the carotid artery of normal rats (Tardif et al., 2015). These data collectively suggest that the greater density of nestin^(+)^-vascular smooth muscle cells may represent an adaptive proliferative phenotype facilitating in part vessel remodeling secondary to hypertension (Table 1). Consistent with a role in vessel remodeling, nestin was expressed in vascular cells of the neointima following balloon injury of the rat carotid artery and in VSMCs identified in human coronary atherosclerotic lesions (Suguta et al., 2007; Oikawa et al., 2010).

## Nestin is induced in pre-existing ventricular cardiomyocytes of the infarcted adult rodent heart

Work from our lab originally identified a modest population of cardiomyocytes at the peri-infarct/infarct region of the ischemically damaged rat heart that expressed nestin in the cytoplasm and/or was partially striated (Table 1; Figure 2) (El-Helou et al., 2005). These findings were independently confirmed and we and others have further reported the appearance of nestin^(+)^-cardiomyocytes in the infarcted human heart (El-Helou et al., 2008; Mokrý et al., 2008; Scobioala et al., 2008; Tamura et al., 2011). Nestin^(+)^-cardiomyocytes detected at the peri-infarct/infarct region co-express desmin, cardiac troponin-T, retain β_1_-adrenergic receptor immunoreactivity, and exhibit a disorganized pattern of connexin-43 expression (Béguin et al., 2009; Meus et al., 2017). The appearance of nestin^(+)^-cardiomyocytes was not a transient response as a population persisted in the heart of 9 month post-myocardial infarcted rats (Béguin et al., 2009). By contrast, nestin immunoreactivity was not identified in ventricular cardiomyocytes of the normal adult heart or non-infarcted region of the ischemically damaged rodent heart (Béguin et al., 2009; El-Helou et al., 2009). Nestin^(+)^-cardiomyocytes were also detected in the adult mouse heart of a genetic model of muscular dystrophy lacking the structural proteins dystrophin and utrophin (Berry et al., 2013). Interestingly, in a pig model of muscular dystrophy, nestin protein levels were robustly increased (33-fold) in skeletal muscle and the intermediate filament protein was re-expressed in regenerating skeletal tissue of mice muscle following denervation (Carlsson et al., 1999; Vaittinen et al., 1999; Fröhlich et al., 2016). Based on the established proliferative role of nestin, recapitulation of the intermediate filament protein in damaged skeletal muscle was considered a phenotypic event of tissue regeneration. In this regard, the appearance of nestin^(+)^-cardiomyocytes in the ischemically damaged heart may likewise represent a regenerative paradigm, albeit the documented modest response precludes a significant impact on infarct size (Meus et al., 2017).

Scobioala et al. (2008) identified morphological small nestin^(+)^-cells co-expressing a panel of putative stem cells markers (e.g., c-Kit, stem cell antigen-1, multiple drug resistance 1, Abcg2) in the infarcted mouse heart. Based on these data, the authors proposed the thesis that the appearance of nestin^(+)^-cardiomyocytes was secondary to the differentiation of resident cardiac stem cells. In the study by Tamura et al. (2011), lineage tracing of neural crest derived cells that displayed a neural progenitor/stem cell phenotype was identified by EGFP staining and a subpopulation apparently differentiated to a cardiomyocyte. A small number of neural crest-derived EGFP^(+)^-cardiomyocytes were detected in the neonatal heart, persisted in the adult myocardium, and identified in the border zone region of the scar following ischemic damage (Tamura et al., 2011). To further explore the aforementioned paradigm, the heart of transgenic mice in which the reporter EGFP was driven by intron 2 of the nestin gene underwent complete coronary artery ligation (Meus et al., 2017). Following ischemic damage, nestin^(+)^-neural progenitor/stem cells that co-expressed EGFP were identified intercalated between cardiomyocytes of the non-infarcted myocardium and detected in the peri-infarct/infarct region (El-Helou et al., 2013; Meus et al., 2017). EGFP staining was absent in nestin^(+)^-cardiomyocytes identified in the infarcted mouse heart demonstrating that cardiac resident neural progenitor/stem cells that express nestin driven by intron 2 of the gene were not the cellular source (El-Helou et al., 2013; Meus et al., 2017). By contrast, cardiomyocyte-specific expression of mCherry directed by α-myosin heavy chain promoter revealed that the *de novo* synthesis of nestin was prevalent in pre-existing mCherry/troponin-T^(+)^-ventricular cardiomyocytes identified at the peri-infarct/infarct region of the ischemically damaged adult mouse heart (Meus et al., 2017) (Table 1). The underlying reason(s) for the discordant findings regarding the cellular source of nestin^(+)^-cardiomyocytes reported by our lab and Tamura et al. (2011) remains presently unresolved.

## Nestin expression in pre-existing cardiomyocytes recapitulates an embryonic phenotype

Lower vertebrates possess the inherent ability to regenerate cardiac tissue following injury (Jopling et al., 2012; Bloomekatz et al., 2016). The adult heart of amphibians and fish contain predominantly small mononucleated cardiomyocytes that contain less myofibrils than adult mammalian cardiomyocytes and retain a proliferative phenotype (Jopling et al., 2012; Bloomekatz et al., 2016). Following cardiac injury, the ventricular regenerative response of lower vertebrates is mediated by the proliferation of pre-existing cardiomyocytes (Jopling et al., 2012; Bloomekatz et al., 2016). Akin to lower vertebrates, growth of the embryonic mammalian heart is mediated by proliferating mononucleated cardiomyocytes (Soonpaa et al., 1996; Brade et al., 2013). The embryonic phenotype of pre-existing mammalian cardiomyocytes is retained for a brief period after birth as exposure of neonatal rodent ventricular cardiomyocytes to a variety of stimuli promote cell cycle re-entry and cytokinesis (Engel et al., 2005; Zebrowski et al., 2016; Meus et al., 2017). Moreover, a cardiac regenerative response was observed following ventricular apex resection of the neonatal mouse heart (Porrello et al., 2011). However, several recent studies have reported that the ventricular regenerative response of the neonatal mouse heart was incomplete or absent (Andersen et al., 2014; Zebrowski et al., 2017). During postnatal (neonatal to adult) growth of the mammalian heart, the embryonic phenotype was lost and the increase in cardiac mass occurred primarily via hypertrophy of binucleated cardiomyocytes derived from mononucleated cardiomyocytes that failed to undergo cytokinesis (Soonpaa et al., 1996; Ahuja et al., 2007). However, it has been suggested that postnatal growth of the rodent and human heart during preadolescence/adolescence may occur in part via the proliferation of a modest population pre-existing cardiomyocytes that retain an embryonic phenotype (Bergmann et al., 2009; Mollova et al., 2013; Senyo et al., 2013; Naqvi et al., 2014). The study by Senyo et al. (2013) reported that throughout the lifespan of a mouse, a very low rate of division of pre-existing cardiomyocytes gives rise to new diploid mononucleated cardiomyocytes. Furthermore, following ischemic damage to the adult mouse heart, the rate of division of pre-existing mononucleated cardiomyocytes was significantly increased leading to the appearance of new cardiomyocytes preferentially at the peri-infarct/infarct region, albeit a ventricular regenerative response was not observed (Senyo et al., 2013). Collectively, these data provided the impetus to assess whether nestin expression in a modest population of pre-existing cardiomyocytes selectively detected at the peri-infarct/infarct region of the adult rodent heart recapitulates an embryonic phenotype. The work by Kachinsky et al. (1995) initially demonstrated that nestin mRNA and protein were expressed in the mouse heart during mid-embryonic development (E9.0–E11) and embryonic cardiomyocytes were nestin immunoreactive. Work from our lab extended the latter findings and further revealed that nestin was detected in atrial and ventricular cardiomyocytes of E9.5 day mice, expression gradually diminished with ongoing cardiac development and was absent at E17.5 days (Meus et al., 2017; Hertig et al., 2018). Nestin was detected in cycling embryonic cardiomyocytes as depicted by Ki67 staining and a subpopulation may have originated from the first heart field characterized by nuclear TBX5 immunoreactivity (Hertig et al., 2018). Filamentous nestin was also identified in H9c2 rat embryonic cardiomyoblasts and lentiviral-short hairpin RNA (shRNA) mediated depletion of the intermediate filament protein significantly inhibited cell cycle re-entry (Meus et al., 2017). Thus, nestin expression recapitulates an embryonic proliferative state and may further provide the requisite phenotype for a subpopulation of pre-existing ventricular cardiomyocytes to potentially re-enter the cell cycle post-MI.

## p38 MAPK suppresses nestin expression and cell cycle re-entry of pre-existing ventricular cardiomyocytes

Despite the appearance of nestin^(+)^-ventricular cardiomyocytes in the infarcted adult rodent heart, the response was modest and cell cycle re-entry was not detected (Béguin et al., 2009; Meus et al., 2017). However, the inherent ability of a subpopulation of pre-existing cardiomyocytes to recapitulate an embryonic phenotype post-MI as reflected by the *de novo* synthesis of nestin would suggest that the modest response and failure to re-enter the cell cycle could be primarily attributed to a suppressive action of one or more biological cues associated with reparative fibrosis. Indeed, the inflammatory response post-MI may play a seminal role inhibiting the cell cycle re-entry of adult ventricular cardiomyocytes in part via activation of the serine/threonine kinase p38 mitogen activated protein kinase (MAPK) (Engel et al., 2005, 2006). Four p38 MAPK isoforms [α, β, γ, and δ] were identified and activation requires the dual phosphorylation of the threonine^180^ (Thr^180^) and tyrosine^182^ (Tyr^182^) residues located in the Thr-Gly-Tyr motif by putative upstream MAPK kinases MKK3 and MKK6 (Arabacilar and Marber, 2015). Adult ventricular cardiomyocytes express p38α/p38β and p38α is the predominant isoform implicated in a variety of biological actions (Arabacilar and Marber, 2015). Two seminal studies by Engel and colleagues revealed that p38α MAPK-dependent pathways inhibit the cell cycle re-entry of embryonic, neonatal and adult cardiomyocytes (Engel et al., 2005, 2006). Furthermore, the co-administration of fibroblast growth factor 1 and the p38 MAPK inhibitor SB203580 to mice subjected to MI led to a smaller scar attributed in part to the cell cycle re-entry and cytokinesis of adult ventricular cardiomyocytes (Engel et al., 2006). Consistent with the latter paradigm, Jopling and colleagues demonstrated that overexpression of p38α MAPK attenuated the regenerative response of the injured zebrafish heart (Jopling et al., 2012). These findings provided the impetus to test the thesis that p38 MAPK may play a seminal role suppressing nestin expression and inhibiting cell cycle re-entry of ventricular cardiomyocytes following ischemic damage to the adult mammalian heart. To examine the latter issue in an *in vitro* setting, neonatal rat ventricular cardiomyocytes (NRVCMs) were employed. In an attempt to mimic cardiac remodeling post-MI, NRVCMs were treated with the protein kinase C (PKC) activator phorbol 12,13 dibutyrate (PDBu). Previous studies have reported that the biological actions of G-protein coupled receptors and cytokines occur in part via the coordinated recruitment PKC- and p38 MAPK-dependent pathways (Bogoyevitch et al., 1994; Schuette and LaPointe, 2000; Pan et al., 2005). Moreover, phorbol ester mediated activation of diacylglycerol-dependent PKC isoforms in ventricular cardiomyocytes and H9c2 rat embryonic cardiomyoblasts led to p38 MAPK phosphorylation (Clerk et al., 1998; Nagarkatti and Sha'afi, 1998). A paucity of NRVCMs isolated from the heart of 1-day neonatal rat pups expressed nestin and exposure to PDBu for 24 h promoted hypertrophy but failed to induce expression of the intermediate filament protein or promote cell cycle re-entry (Meus et al., 2017). An analogous paradigm was observed in the rat heart secondary to hypobaric hypoxia as nestin expression was not upregulated in the hypertrophied right ventricle (Chabot et al., 2016). PDBu treatment of NRVCMs led to p38 MAPK phosphorylation and phosphorylation of the serine^82^ residue of the downstream target heat shock protein 27 (HSP27) (Meus et al., 2017). Inhibition of p38 MAPK activity with the pharmacological agent SB203580 suppressed PDBu-mediated phosphorylation of the serine^82^ residue of HSP27 (Meus et al., 2017). The 24 h treatment of NRVCMs with PDBu and SB203580 induced nestin expression and promoted cell cycle re-entry and the response was potentiated following a 72 h exposure (Table 1) (Meus et al., 2017; Hertig et al., 2018). By contrast, SB203580 did not inhibit the hypertrophic response of NRVCMs in response to PDBu (Meus et al., 2017; Hertig et al., 2018). Lastly, nestin depletion with an AAV9 containing a shRNA directed against the intermediate filament protein significantly attenuated the number of NRVCMs that expressed nestin and re-entered the cell cycle (Table 1) (Hertig et al., 2018). Collectively, these data reveal that nestin-driven cell cycle re-entry of pre-existing cardiomyocytes may represent a physiological response during reparative fibrosis potentially highlighting an inherent paradigm of ventricular regeneration. Indeed, the smaller infarct reported in the rat heart secondary to isoflurane preconditioning as compared to the non-preconditioned infarcted heart was associated with a significant increase in the appearance of nestin^(+)^-ventricular cardiomyocytes (Agnić et al., 2015). However, in the infarcted adult mammalian heart, recruitment of p38 MAPK secondary to the overt inflammatory response may represent a seminal event limiting the appearance of nestin^(+)^-cardiomyocytes and the concomitantly inhibiting cell cycle re-entry.

## Nestin expression and cell cycle re-entry of neonatal ventricular cardiomyocytes requires the recruitment of Yap-1 and PKB

HIPPO-regulated transcriptional coactivator yes associated protein-1 (Yap-1) plays a seminal in cardiac embryogenesis as dephosphorylation promotes cardiomyocyte proliferation leading to a thickened compact myocardium and expanded trabecular layer (Xin et al., 2013). Furthermore, dysregulation of the HIPPO pathway secondary to the depletion of the scaffold protein Salavador led to the nuclear accumulation of dephosphorylated Yap-1 in adult cardiomyocytes initiating cell cycle re-entry and ventricular regeneration following an ischemic insult (Heallen et al., 2013). Consistent with the latter premise, the absence of nestin expression and cell cycle re-entry of NRVCMs following PDBu treatment alone was associated with Yap-1 inactivation following phosphorylation of the serine^127^ residue (Hertig et al., 2018). Thus, at least in response to PDBu, Yap-1 was dispensable in the hypertrophic response of NRVCMs (Hertig et al., 2018). Yap-1 does not represent a direct target of p38 MAPK suggesting that phosphorylation of the transcriptional coactivator may have occurred at least in part via recruitment of upstream kinases Mammalian sterile 20-like kinase 1 (Mst1) and/or large tumor suppressor 1/2 (Lats1/2) (Lee and Yonehara, 2012). PDBu/SB203580 co-treatment of NRVCMs led to the dephosphorylation of the serine^127^ residue of Yap-1 and verteporfin inhibition of dephosphorylated Yap-1 signaling suppressed nestin expression and cell cycle re-entry (Hertig et al., 2018). A recent study revealed that Yap-1 facilitated cardiomyocyte proliferation via recruitment of phosphatidylinositol-3 kinase/phosphatidylinositol-3 kinase dependent kinase/protein kinase B (PI3-K/PDK/PKB)-dependent pathway (Lin et al., 2015). Nestin mediated cell cycle re-entry of normal and tumorigenic cells and growth of the embryonic heart following overexpression of the intermediate filament protein occurred via a PI3-K/PDK/PKB-dependent pathway (Xue and Yuan, 2010; Chen et al., 2014; Liu et al., 2015). Moreover, PKB was identified as a key downstream signaling event facilitating phosphatidylinositol-3 kinase mediated cell cycle re-entry of embryonic and adult cardiomyocytes (Evans-Anderson et al., 2008; Beigi et al., 2013; Lin et al., 2015). These observations suggest that nestin may drive Yap-1 mediated cell cycle re-entry of NRVCMs via recruitment of phosphatidylinositol-3 kinase dependent signaling events. To address the latter premise, NRVCMs were treated with the pharmacological agent triciribine reported to prevent phosphorylation/activation of PKB by inhibiting binding to the pleckstrin homology domain of PDK (Berndt et al., 2010). Triciribine administration to PDBu/SB203580-treated NRVCMs inhibited BrdU incorporation but concomitantly increased the number of NRVCMs that expressed nestin (Hertig et al., 2018). Thus, at least in response to PDBu/SB203580, Yap-1 signaling selectively induces nestin expression, and the intermediate filament protein acting via recruitment of PKB promotes cell cycle re-entry of NRVCMs. Moreover, the increase in the number of nestin^(+)^-NRVCMs following PDBu/SB203580 and tricirbine co-treatment is consistent with previous studies demonstrating that PKB directly phosphorylates/inactivates Yap-1 (Basu et al., 2003; Zhang et al., 2012).

## Conclusion

Cardiac resident nestin^(+)^-neural progenitor/stem cells identified in the normal adult rodent heart represent a cellular substrate of *de novo* blood vessel formation during reparative fibrosis. However, the relative contribution of nestin^(+)^-neural progenitor/stem cells to the reparative angiogenic response during scar formation and healing remains presently undefined. The biological impact attributed to the increased density of nestin/collagen type I^(+)^-fibroblasts is dependent on whether the underlying context involves reparative or reactive fibrosis. Thus, at least in the absence of ischemic damage, targeting nestin expression in ventricular fibroblasts of the pressure-overloaded heart and other fibrotic tissue (e.g., lungs, kidney) may represent a potential therapeutic approach to attenuate reactive fibrosis. Recapitulation of an embryonic phenotype and seminal role of the intermediate filament protein driving the cell cycle re-entry of neonatal cardiomyocytes and cardiomyoblasts suggests that the appearance of a modest number of pre-existing nestin^(+)^-adult ventricular cardiomyocytes post-MI may represent an inherent paradigm of ventricular regeneration. However, the limited appearance of nestin^(+)^-ventricular cardiomyocytes and concomitant inability to re-enter the cell cycle may be attributed in part to the recruitment of p38 MAPK secondary to the overt inflammatory response post-MI. Pharmacologically targeting p38 MAPK or identifying downstream targets of the serine/threonine kinase that selectively suppress expression of the intermediate filament protein may represent a therapeutic approach to initiate a partial cardiac regenerative response post-MI secondary to the increased appearance of cycling nestin^(+)^-ventricular cardiomyocytes. Lastly, vascular remodeling secondary to hypertension was attributed in part to the increased density of nestin^(+)^-vascular smooth muscle cells the re-entered the cell cycle. Nestin upregulation in VSMCs may represent an adaptive proliferative response driving in part remodeling of the vasculature secondary to a chronic hemodynamic overload (Briet and Schiffrin, 2013).

## Author contributions

The author confirms being the sole contributor of this work and approved it for publication.

### Conflict of interest statement

The author declares that the research was conducted in the absence of any commercial or financial relationships that could be construed as a potential conflict of interest.
